# Species delimitation, genetic diversity and population historical dynamics of *Cycas diannanensis* (Cycadaceae) occurring sympatrically in the Red River region of China

**DOI:** 10.3389/fpls.2015.00696

**Published:** 2015-09-08

**Authors:** Jian Liu, Wei Zhou, Xun Gong

**Affiliations:** ^1^Key Laboratory for Plant Diversity and Biogeography of East Asia, Kunming Institute of Botany, Chinese Academy of SciencesKunming, China; ^2^Key Laboratory of Economic Plants and Biotechnology, Kunming Institute of Botany, Chinese Academy of SciencesKunming, China; ^3^University of Chinese Academy of SciencesBeijing, China; ^4^Yunnan Key Laboratory for Wild Plant ResourcesKunming, China

**Keywords:** *Cycas*, species delimitation, tree-based, sympatry, population dynamics, conservation genetics, Red River region, China

## Abstract

Delimitating species boundaries could be of critical importance when evaluating the species' evolving process and providing guidelines for conservation genetics. Here, species delimitation was carried out on three endemic and endangered *Cycas* species with resembling morphology and overlapped distribution range along the Red River (Yuanjiang) in China: *Cycas diananensis* Z. T. Guan et G. D. Tao, *Cycas parvula* S. L. Yang and *Cycas multiovula* D. Y. Wang. A total of 137 individuals from 15 populations were genotyped by using three chloroplastic (*psb*A-*trn*H, *atp*I*-atp*H, and *trn*L*-rps*4) and two single copy nuclear (*RPB*1 and *SmHP*) DNA sequences. Basing on the carefully morphological comparison and cladistic haplotype aggregation (CHA) analysis, we propose all the populations as one species, with the rest two incorporated into *C. diannanensis*. Genetic diversity and structure analysis of the conflated *C. diannanensis* revealed this species possessed a relative lower genetic diversity than estimates of other *Cycas* species. The higher genetic diversity among populations and relative lower genetic diversity within populations, as well as obvious genetic differentiation among populations inferred from chloroplastic DNA (cpDNA) suggested a recent genetic loss within this protected species. Additionally, a clear genetic structure of *C. diannanensis* corresponding with geography was detected based on cpDNA, dividing its population ranges into “Yuanjiang-Nanhun” basin and “Ejia-Jiepai” basin groups. Demographical history analyses based on combined cpDNA and one nuclear DNA (nDNA) *SmHP* both showed the population size of *C. diannanensis* began to decrease in Quaternary glaciation with no subsequent expansion, while another nDNA *RPB1* revealed a more recent sudden expansion after long-term population size contraction, suggesting its probable bottleneck events in history. Our findings offer grounded views for clarifying species boundaries of *C. diannanensis* when determining the conservation objectives. For operational guidelines, the downstream populations which occupy high and peculiar haplotypes should be given prior in-situ conservation. In addition, ex-situ conservation and reintroduction measures for decades of generations are supplemented for improving the population size and genetic diversity of the endemic and endangered species.

## Introduction

The conceptualization and boundary of species are critically important and of great significance for taxonomists, ecologists and conservation biologists when identifying objective taxa and determining the protection units. However, the issue “what a species is” that has long been debated since Darwin's time is still controversial (Dobzhansky and Dobzhansky, 1937; Mayr, 1942; Mallet, 1995; de Queiroz, 2005; De Queiroz, 2007), with none such a unified definition being generally accepted. As the raising concerns on the topic of speciation (Turelli et al., 2001; Wu, 2001; Coyne and Orr, 2004; Lexer and Widmer, 2008; Butlin et al., 2012) in recent decades, species delimitation again attracts evolution biologists' attention (Wiens and Penkrot, 2002; Wiens, 2007; Petit and Excoffier, 2009; Carstens and Dewey, 2010; Fujita et al., 2012) and specific models and methodologies were put forward by employing morphological characters (Wiens and Penkrot, 2002), genetic datasets (O'meara, 2009; Yang and Rannala, 2010; Barrett and Freudenstein, 2011; Ence and Carstens, 2011; Harrington and Near, 2012; Niemiller et al., 2012), or geographical data (Rissler and Apodaca, 2007) to clarify the lineage's speciation process and to delimit species. *Cycas* L. (Cycadaceae), which is considered as the basal lineage of the Cycadales, and also the sister group to the other gymnosperms (Burleigh et al., 2012; Lu et al., 2014; Wang and Ran, 2014), contains approximately 105 extant species around the world (Haynes, 2011), mainly distributed in the tropic and sub-tropic areas around the Pacific. As an endangered but quite recent (mid-Miocene) radiant gymnosperm genus (Nagalingum et al., 2011), phenotype variations can not necessarily assort *Cycas* into discrete categories. As a result, some morphology-resembled or character-equivocal species due to interspecific hybridization were often put forward by a blended name of “complex” or “group” (see Hill, 1994a,b; Yang and Meerow, 1996; Liu, 2004; Xiao and Gong, 2006), making the definition of a species impeded to botanical studies of speciation.

The Red River origins in northwest Yunnan of China, and is named as “Yuanjiang” in the basin of Yunnan, then flows through southwest Yunnan and northern Vietnam and out to the Gulf of Tonkin. The basin of the Red River corresponds to a geological fault zone (Red River fault zone, RRFZ) that is resulted from the uplifting of Himalaya and the basin expansion of South China Sea (Harison et al., 1992; Leloup et al., 1995). The RRFZ stretches for more than 1000 km on land which stands out a discontinuity in the geology of Yunnan (Tapponnier et al., 1990), and harbors an abundant *Cycas* diversity with more than 14 species, in which 10 are endemic to this region (Hill, 2008). Within these species, *C. diannanensis, C. parvula*, and *C. multiovula* are three sympatric and morphological related species which are all classified into the Section *Stangeriodes* by sharing glabrous ovules, soft microsporangiate sporophylls and yellow seeds. The three species also display similar un-subterraneous stem habit and long cataphylls which made it difficult to identify them when no reproductive organ exists. The morphological differences between *C. diannanensis* and *C. parvula* are in the shape of megasporophyll terminal lamina, with the former one possessing broader terminal lamina while the terminal lamina of *C. parvula* is pinnately parted. For *C. diannanensis* and *C. multiovula*, they only differ in the number of ovules and the size of megasporophyll, with the later normally owning more ovules and larger megasporophyll.

The classification of *Cycas* in China is confused especially after numerous disputable new species being described since the 1990s (Wang et al., 1996; Wu and Raven, 1999; Hill, 2008). None of the above three species is listed in *Flora of China* (Wu and Raven, 1999), in which they are treated as synonyms of other species, and only *C. diannanensis* is accepted by the world list of cycads (Haynes, 2011). Previous studies held different opinions when dealing with the issue whether the three similar but controversial species could be good species. Jiang (2004) considered the other two species should be incorporated into *C. diannanensis* based on his wild survey and morphological comparisons with specimens. However, Nong et al. (2011) thought *C. parvula* should be independent species according to their RAPD results, although in their study *C. parvula* was clustered with *C. diannanensis*. Some other results based on palynology (Wang, 2000) and ISSR data (Xiao and Gong, 2006) also considered *C. parvula* should be a good species, whereas in their studies the sympatric *C. diannanensis* and *C. multiovula* were absent of sampling for comparison. Moreover, the samples and genetic markers in these studies were limited, since these factors have great impacts on the results when delimiting species (Knowles and Carstens, 2007). Therefore, subsequent taxonomical revision and phylogenetic analysis are required to clarify whether these three species could be good species respectively. Meanwhile, under the circumstance of wild populations' severe situation of the *Cycas* species through the investigations along the Red River, as well as the urgent threatening status of *Cycas* species in China, it should be impending to determine the actual species boundaries and evaluate the genetic diversity basing on comprehensive sampling and different molecular approaches to carry out reasonable protection strategies for them.

The geographical distribution of plant species had been profoundly influenced by the climatic oscillations in the Quaternary (Hewitt, 2000), and species colonization or contraction triggered by such climate fluctuations may lead to unexpected genetic subdivision and mixture of populations (Comes and Kadereit, 1998; Hewitt, 2004). The genetic structure of existing populations can be imprinted by historical processes (e.g., ice age), especially for those long-evolved and sessile organisms (Feng et al., 2014). Genetic data can provide insights into adaptive potential for particular species in postglacial colonization refugia as well as valuable information and suggestions for the species delimitation, demographic history and conservation categories (Gong et al., 2011; Zhao and Gong, 2012; Jia et al., 2014). In this study, we sequenced three maternally inherited cpDNA and two biparentally inherited nDNA markers of 15 populations from *C. diannanensis, C. parvula*, and *C. multiovula*, which shared an overlapped distribution in the Red River basin, and examined the genetic relationships between them. In doing so, we aim to demarcate the boundaries among these sympatric species, then evaluate the genetic diversity, genetic structure and demographic history of the existing populations, and ultimately provide valid conservation guidelines for the ancient and endangered species.

## Materials and methods

### Taxon sampling

A total of 137 individuals were selected for subsequent analysis from the 15 populations collected along the Red River region in China, including 10 populations of *C. diannanensis*, three populations of *C. parvula* and two populations of *C. multiovula* (sampling 10 individuals for each population and all individuals for populations less than 10). Among them, materials of *C. parvula* and *C. multiovula* were obtained from cultivated individuals in the village after the verification that they were from the same population. The *C. diannanensis* population ZSM and *C. multiovula* population ZSD were sampled from the same place (Zhongshan, Chuxiong). Information of sampling sites and the number of individuals from each population used in this study are presented in Table 1.

**Table 1:** **Details of sampling of the ***Cycas*** populations investigated in this study**.

**Species**	**Sampling location**	**Population code**	**Latitue (N^0^)**	**Longtitude (E^0^)**	**Altitude (m)**	**Selected (and collected) Individuals**
*C. parvula*	Huashiban, Yuanjiang	YJH	23.552	101.926	1100	10(11)
	Majie, Yuanyang	YYM	23.259	102.662	1200	7(7)
	Dong'e, Yujiang	DEY	23.703	101.782	1100	10(28)
*C.multiovula*	Zhongshan, Chuxiong	ZSD	24.807	101.987	1700	5(5)
	Gejiu, (in downtown)	GJD	23.359	103.160	1700	5(5)
*C. diannanensis*	Dutian, Chuxiong	DTX	24.532	101.465	2000	10(10)
	E'jia, Chuxiong	EJT	24.505	101.243	1500	10(28)
	Wotuodi, Shuangbai	EJW	24.546	101.207	900	10(26)
	Hongtupo, Nanhua	HTP	24.945	101.892	1700	10(27)
	Xinqiao, Xinping	JPX	22.865	103.571	2000	10(22)
	Manhao,Gejiu	MHG	23.019	103.413	1200	10(28)
	Gasa, Xinping	XPG	24.044	101.530	1500	10(22)
	Da'me,Chuxiong	XSD	24.721	101.017	1350	10(15)
	Yisha, Chuxiong	XSY	24.643	101.085	1000	10(26)
	Zhongshan, Chuxiong	ZSM	24.807	101.987	1000	10(15)

*All locations are situated along the Red River in Yunnan, China*.

### DNA extraction, PCR amplification, sequencing and cloning

Materials for DNA extraction were from young and healthy leaves which were collected and dried immediately in silica gel. Genomic DNA was extracted from dried leaves using the modified CTAB method (Doyle, 1991). Approximately 2–3 individuals from each population were selected for preliminary screening from universal chloroplastic and nuclear primers. A total of five markers were selected and sequenced within the total 137 individuals, including three cpDNA intergenic spacers: *psb*A-*trn*H, *rps*4*-trn*L, and *atp*I*-atp*H (Shaw et al., 2005), and two single copy nuclear genes: *Cycas revoluta* RNA polymerase II largest subunit, *RPB*1 and *Selaginella moellendorffi* hypothetical protein, *SmHP* (Chiang, Y. C., unpublished) for complete analysis (for primer information, see Table 2). PCR amplification was carried out in 40 μL volume reactions. For cpDNA, the PCR reactions contained 20 ng DNA, 2.0 μL MgCl_2_(25 mM), 2.0 μL dNTPs (10 mM), 4.0 μL 10 × PCR buffer, 0.6 μL of each primer, 0.6 μL Taq DNA polymerase (5 U/μL) (Takara, Shiga, Japan) and 26 μL double-distilled water. For nDNA, the PCR reactions contained 40 ng DNA, 2.4 μL MgCl_2_ (25 mM), 2.0 μL dNTPs (10 mM), 2.0 μL DMSO, 4.0 μL 10 × PCR buffer, 0.7 μL of each primer, 0.7 μL Taq DNA polymerase (5 U/μL) (Takara, Shiga, Japan) and 24.6 μL double-distilled water. PCR amplifications were performed in a thermocycler under the following conditions: an initial 5 min denaturation at 80°C, followed by 34 cycles of 1 min at 95°C, 1 min annealing at 50°C, and a 1.5 min extension at 65°C, and a final extension for 10 min at 65°C for cpDNA intergenic spacers. For nDNA sequences, an procedure of initial 4 min denaturation at 94°C, which was followed by 34 cycles of 50 s at 94°C, 1 min annealing at 50°C (for *SmHP*) or 55°C (for *RPB*1), and a 1.5 min extension at 72°C, and a final extension for 10 min at 72°C was used. All PCR products were sequenced in both directions with the same primers for the amplification reactions, using an ABI 3770 automated sequencer at Shanghai Sangon Biological Engineering Technology & Services Company Ltd. The individuals with nDNA sequences which had one or more heterozygous sites in the first sequencing round were subsequently cloned. PCR products were purified using the TIANgel Midi Purification Kit (Tiangen). Purified products were linked to pMD18-T Vector and then inserted to *E. coli DH5*α strains. Six to ten clones were randomly picked and sequenced until the heterozygous site split into two alleles. The data sets of the DNA sequencing in this study were deposited in GenBank (accession numbers from KT334601–KT334653).

**Table 2:** **cpDNA and nDNA fragments and primer sequences used in this study**.

**Region**	**Primer sequences (5′- sequence - 3′)**	**References**
*psb*A – *trn*H (cpDNA)	*psb*A: GTT ATG CAT GAA CGT AAT GCT C *trn*H: CGC GCA TGG TGG ATT CAC AAT CC	Shaw et al., 2005
*rps*4 – *trn*L (cpDNA)	*rps*4: CTG TNA GWC CRT AAT GAA AAC G *trn*L: TCT ACC GAT TTC GCC ATA TC	Shaw et al., 2005
*atp*I – *atp*H (cpDNA)	*atp*I : TAT TTA CAA GYG GTA TTC AAG CT *atp*H: CCA AYC CAG CAG CAA TAA C	Shaw et al., 2005
*RPB1* (nDNA)	F010: GTA CCC CAG TCA TTT GAG AC R1142: AGC CAG CAG TAA CCA TTG CC	In this study
*SmHP* (nDNA)	F004: CAA AAC TAT GCT GTC AAT CC R745: TTA GCA TCA CCA GTA ATC CC	In this study

### Data analysis

The cpDNA and nDNA sequences were edited and generated by SeqMan (Swindell and Plasterer, 1997). Multiple alignments of the DNA sequences were manually refined with Clustal X v1.83 (Thompson et al., 1997), with subsequent adjustment in Bioedit v7.0.4.1 (Hall, 1999). Although the congruency test for the three combined cpDNA regions in this study showed a non-significant rate of homogeneity (*P* = 0.4, < 0.5) by PAUP^*^ 4.0b10 (Swofford, 2002), suggesting indistinctive degree of homogeneity between the cpDNA regions, we still combined these three regions to gain enough variable sites in the subsequent analysis as some former studies suggested (Yoder et al., 2001; Quicke et al., 2007).

Haplotypes from five markers for all the three species were calculated from aligned DNA sequences by DnaSP v5.0 (Librado and Rozas, 2009). The genetic diversity within- and among-populations were estimated by calculating Nei's nucleotide diversity (Pi) and haplotype diversity (*Hd*) indices through DnaSP software as well. The within-population gene diversity (*H*_*S*_), gene diversity in total populations (*H*_*T*_) and two coefficient of population differentiation, *G*_*ST*_ and *N*_*ST*_ were calculated by Permut v1.0 (http://www.pierroton.inra.fr/genetics/labo/Software/Permut).

The DnaSP v5.0 software was also used to investigate the demography of the species and check if the evolution matched with neutral mutation. The Tajima's *D* and Fu and Li's F^*^ value were calculated to detect departures from population equilibrium, and the pairwise mismatch distribution was used to test for population expansion. We also used Arlequin v3.0 (Excoffier et al., 2005) to calculate the raggedness index and its significance to quantify the smoothness of the observed mismatch distribution. The sum-of-squared deviations (SSD) between the observed and expected mismatch distributions were computed, and *P*-values were calculated as the proportion of simulations producing a larger SSD than the observed SSD. The relatedness degree among cpDNA and among nDNA haplotypes were estimated by using Network v4.2.0.1 (Forster et al., 2007). In the network analysis, we treated an indel as one single mutational event. The Arlequin v3.0 (Excoffier et al., 2005) was used to conduct an analysis of molecular variance (AMOVA) and to estimate the genetic variation assigned within and among populations. Isolation by distance (IBD) model was tested between all pairs of populations by computing Mantel tests in GenAlEx package version 6.3 (Peakall and Smouse, 2006) using a correlation between F_ST_ and geographic distance.

Phylogenetic relationships among cpDNA and nDNA haplotypes generated from the three species were inferred using maximum likelihood method by online PhyML (http://www.atgc-montpellier.fr/phyml/) (Guindon et al., 2010) and Bayesian inference by MrBayes v3.2 (Ronquist et al., 2012), in which we employed a distinct species *Cycas tanqingii* D. Y. Wang as outgroup. We referred a tree-based species delimitation method of cladistic haplotype aggregation (CHA, Brower, 1999) which tabulated the testing haplotypes to determine the population profiles, and aggregated haplotypes that sharing identical profiles, then estimated the phylogeny of the unaggregated groups of haplotypes, and divided sets of topologically contiguous populations into separate species. The divergence time between lineages within populations were estimated by BEAST v1.7 (Drummond et al., 2012) with a strict molecular clock and the evolutionary rates set as 1.01 × 10^−9^ and 5.1–7.1 × 10^−9^ (6 × 10^−9^ in this study) mutation per site per year for cpDNA and nDNA respectively, which had previously been estimated in seed plants for synonymous sites (Graur and Li, 2000). The time of the basal node inferred from the average evolutionary rate was used as an age constraint for earliest lineage divergence. The phylogenic relationship of all samples was also constructed by MrBayes v3.2 (Ronquist et al., 2012) to infer the individuals' clustering for species delimitation, in which four simultaneous runs with four chains each were run for combined data for 10^7^ generations and trees were sampled every 1000 generations, with the first 25% trees of the sample trees from each run were discarded. The above sampling data after Bayesian analysis was examined and determined by Tracer v1.6 (Rambaut et al., 2014). Before the phylogenetic analysis, the best evolution models were chosen by jModeltest 1.7 (Posada, 2008; Darriba et al., 2012) for both combined cpDNA (F81+G for AIC, F81 for BIC) and nDNA (both HKY+I for two sequences).

A Bayesian Skyline plot was also calculated by the BEAST v1.7 (Drummond et al., 2012) to infer the historical demography of species in this study. Posterior estimates of the mutation rate and time of divergence were obtained by Markov Chain Monte Carlo (MCMC) analysis. The analysis was run for 10^7^ iterations with a burn-in of 10^6^ and a strict clock model under the HKY+I evolution model for both cpDNA and nDNA. Genealogies and model parameters were sampled every 1000 iterations. Convergence of parameters and mixing of chains were followed by visual inspection of parameter trend lines and checking of effective sampling size (ESS) values in three pre-runs. The ESS parameter was expected to surpass 200, which suggested acceptable mixing and sufficient sampling in analysis. Adequate sampling and convergence to the stationary distribution were checked using Tracer v1.6 (Rambaut et al., 2014).

We also conducted an analysis on both population structure and species delimitation by the sequence data using STRUCTURE v2.2 (Evanno et al., 2005), as strategy employed by STRUCTURE is straightforward and matches reasonably well the properties of metapopulation lineages (Shaffer and Thomson, 2007). Sequence data were first converted to structure format. Ten independent runs were performed for each set, with values of K ranging from 1 to 15, a burn-in of 1 × 10^5^ iterations and 1 × 10^5^ subsequent MCMC steps. The combination of an admixture and a correlated-allele frequencies model was used for the analysis. The best-fit number of grouping was evaluated using ΔK by STRUCTURE HARVESTER, v0.6.8 (Earl, 2012).

## Results

### DNA sequences characterization

**Figure 1:**
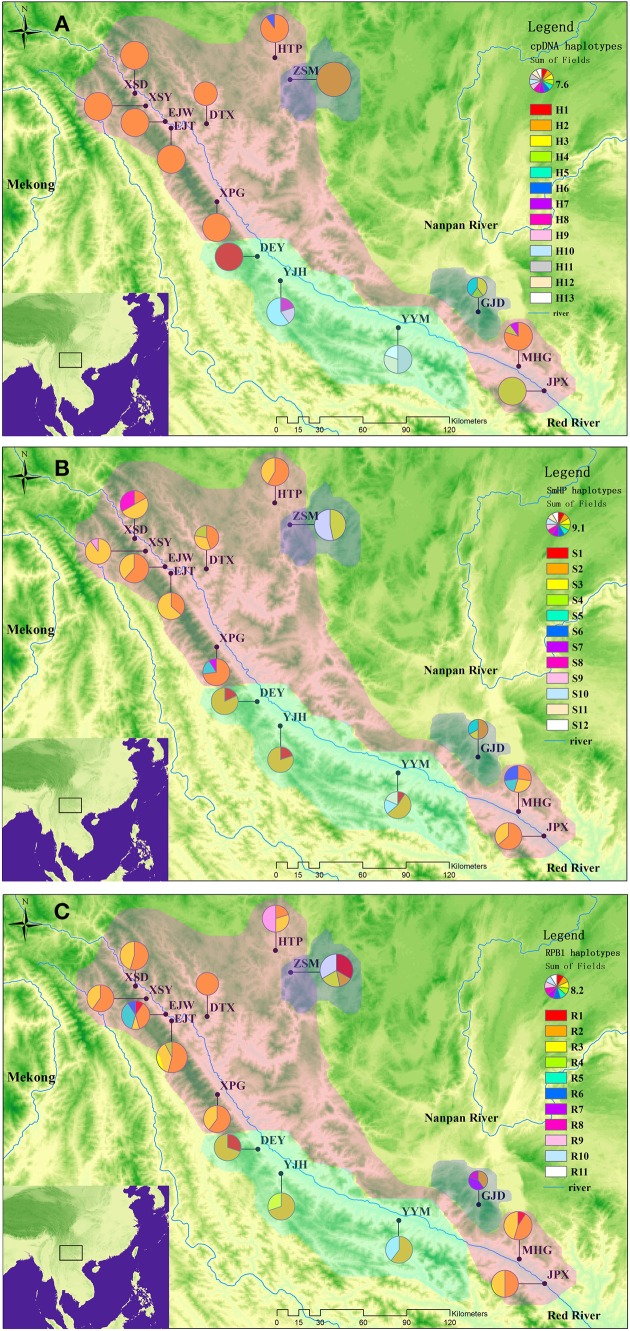
**Geographical distributions of cpDNA haplotypes (A), nDNA ***SmHP*** haplotypes (B), nDNA ***RPB***1 haplotypes (C) in *C. diannanensis***. Frequencies of haplotypes in each population are indicated by the proportions of pie diagrams. The colored dash areas present the sampling and possible distributions of the *Cycas* species in this study: Red, *C. diannanensis*; Green, *C. parvula*; Blue, *C. multiovula*.

The combined chloroplastic sequence data of *atp*I*-atp*H, *psb*A-*trn*H, and *trn*L-*rps*4 was aligned as a consensus length of 1992 bp, containing 61 polymorphic sites among which 14 were substitutions and others were indels. A total of 13 haplotypes were detected in the 15 populations. The haplotype distributing patterns were listed in the Table S1 and showed in the Figure 1A.

The single nuclear copy gene *SmHP* (F004-R745) sequence matrix was aligned with a consensus length of 664bp, which contained 15 substitution sites, and formed 12 nuclear haplotypes in the 15 populations. The most widely distributed haplotypes were Hap S2 and S3, which occurred in 12 and 10 populations respectively and were shared by all the three species (Figure 1B).

The other nDNA *RPB*1 (010-1142) sequence matrix was aligned with an accordant length of 912bp, in which six substitutions were detected, deriving 11 nuclear haplotypes in total (Figure 1C).

### Network analysis

**Figure 2:**
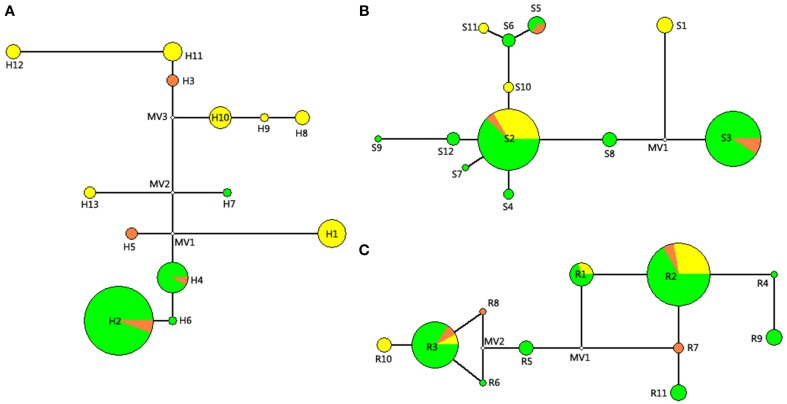
**Networks of the combined cpDNA sequence (A), nDNA ***SmHP*** (B), and nDNA ***RPB***1 (C) haplotypes of studied ***Cycas*** species**. Each circle represents one haplotype. The size of the circles corresponds to the frequency of each haplotype, and the small hollow circles indicate hypothetical missing haplotype. For different colors in this figure: Green, *C. diananensis*, yellow, *C. parvula*, orange, *C. multiovula*.

For the network diagram of combined cpDNA (Figure 2A), three missing haplotypes occurred in the internal nodes. The ancestral haplotype was also missed, with the low frequent haplotypes locating at the external position of the network diagram besides haplotype H2. Each haplotype in the cpDNA network kept one nucleotide difference to the nearest haplotype except haplotype H11 with H12 (six variations) and haplotype H1 with MV1 (missing haplotype, three steps).

For network analysis of nDNA *SmHP* sequence (Figure 2B), one missing haplotype was detected and the highest frequent haplotype S2 was shared by all the species and located at the center position of the reticulate evolution diagram, suggesting Hap S2 as the ancestral haplotype. For *RPB*1 gene, two loops and two missing haplotypes occurred in the network diagram. All the haplotypes held one nucleotide difference with its adjacent haplotype. Haplotype R5 located in the center position of the network diagram with most others placing at the external nodes (Figure 2C).

### Haplotype phylogeny (aggregation), divergence and species clustering analysis

**Figure 3:**
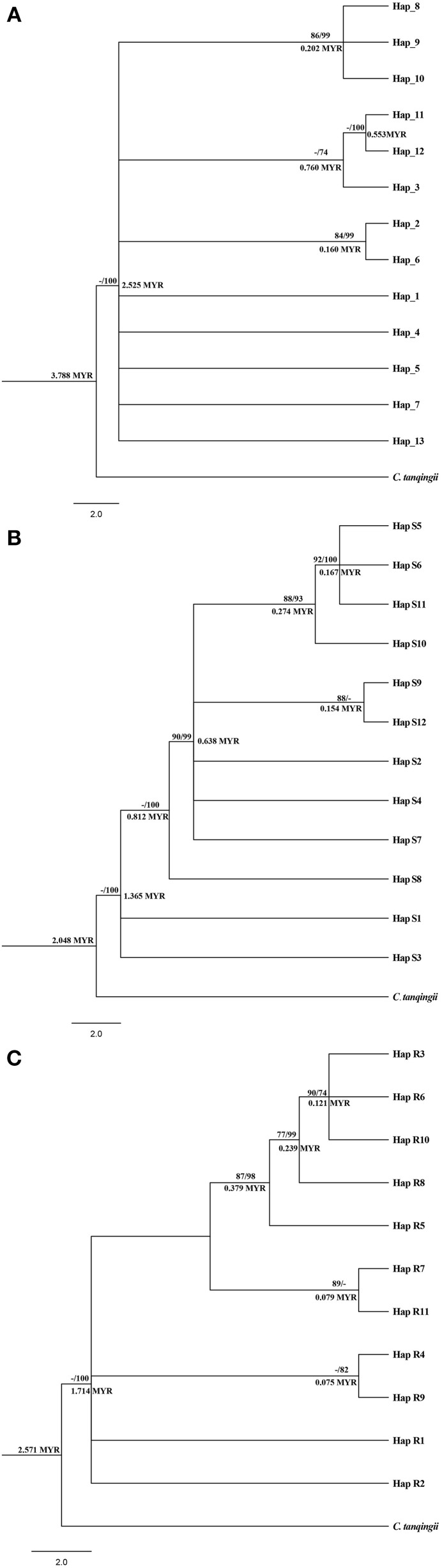
**Phylogenetic analysis and divergent time obtained from cpDNA haplotypes (A), nDNA ***SmHP*** haplotypes (B), and nDNA ***RPB***1 haplotypes (C) of the three ***Cycas*** species**. Number above the line of each note stands for the bootstrap value of Maximum Likelihood/and posterior probability (*PP*) inferred from Bayesian inference (for *PP* > 70). Number below the line represents divergent time by BEAST v1.7. MYR: million years.

Maximum likelihood (ML) analysis and Bayesian inference of cpDNA and nDNA haplotypes generated similar cladograms corresponding to the network analysis, whereas differed in the support values located on internal nodes. For cpDNA, all 13 haplotypes appeared as a distinct comb-like structure with three paraphyletic subclades nested inside. Within the cladogram, haplotypes H12, H11, and H3 clustered in the same subclade which was occupied by *C. parvula* and *C. multiovla* from the downstream populations YYM and GJD, implying these two adjacent populations should be closely related. For the other subclades, the most widely distributed H2 was clustered with the haplotype H6, which was specific by upstream population HTP. Haplotype H8, H9 as well as H10 which were all peculiar in YJH shared a close relationship to form a subclade, whereas this subclade was nonexclusive with other haplotypes in the large clade (Figure 3A). As the evidence that none of the above lineage inferred from cpDNA data could be separated from all other populations by a branch in the cladogram, nor could they form a contiguous section in the network analysis (Figure 2), we deduced all the lineages (haplotypes) as one phylogenetic species.

For nDNA *SmHP*, the first divergent haplotypes were Hap S1, S3, and S8, which were widely shared by all the populations (Table S1, Figure 3B), suggesting these three haplotypes (especially for S1 and S3) were more ancient than others. The other haplotypes formed one clade, within which the relationship were not fully resolved (Figure 3B). For nDNA *RPB*1, haplotype R1 and R2 were first divergent haplotypes from MV1 (missing haplotype 1) which was mapped in middle of the network evolution diagram. The second most frequent haplotype Hap S3 located at the top of the haplotype cladogram and the near margin of network evolution diagram, suggesting it a recent evolved haplotype. Similarly, haplotypes aggregation of *RPB1* in each branch (clade) from the cladogram neither matched with the species populations nor geographical distributions (Table S1, Figure 3C).

Inference of divergent time of haplotypes from our cpDNA and nDNA data all suggested a recent divergence of the *Cycas* lineages (for cpDNA: 3.788 Myr (million years), *SmHP*: 2.048 Myr, *RPB1*: 2.571 Myr), indicating a haplotype splitting in late Pliocene (Piacenzian, 3.6 Myr) or within Pleistocene (2.6 Myr). Estimate time of different haplotypes on the internal divergence node was displayed on Figure 3.

All three phylogenic trees (combined cpDNA, *SmHP* and *RPB1*) showed well supported lineage clades (most *PP* > 90) of the three species (Figure S1). Nevertheless, none of the phylogram could explicitly generated monophyletic clade within each morphological identified species. Individuals from the *C. parvula* populations were located at the basal clades inferred from cpDNA data, and the other two species from different populations displayed non-aggregated clustering which were also conflicted to morphological classification through both cpDNA and nDNA data.

### Genetic diversity and genetic structure

Relative lower total nucleotide (Pi) and haplotype (*Hd*) diversity in all populations were detected in combined cpDNA (0.00087 and 0.564, respectively) than in nDNA (*Pi* = 0.00471, *Hd* = 0.67 for *SmHP*; *Pi* = 0.00302, *Hd* = 0.671 for *RPB*1, see Table S1). Total genetic diversity (*H*_*T*_ = 0.627 for cpDNA, 0.667 for *RPB*1, 0.679 for *SmHP*) was higher than the average intra-population diversity (*H*_*S*_ = 0.179, 0.562, 0.528 from cpDNA, *RPB1* and *SmHP* respectively, Table 3), resulting in overall high level of genetic differentiation within populations (*F*_*ST*_ = 0.819, 0.055, 0.251 from cpDNA, *RPB*1 and *SmHP*, respectively). For cpDNA, most populations displayed no haplotype diversity except population GJD, MHG, HTP, YJH, and YYM, which distributed along downstream of the Red River. However, most populations occupied high haplotype diversities at the nDNA level.

**Table 3:** **Genetic diversity, differentiation parameters for the combined chloroplast DNA (cpDNA) sequences and two nuclear loci (***SmHP, RPB1***) in all population of ***C. diannanensis*****.

**Locus**	***H_*T*_***	***H_*S*_***	***G_*ST*_***	***N_*ST*_***
cpDNA	0.627 (0.147)	0.192 (0.080)	0.694 (0.093)	0.836 (0.057)
*SmHP*	0.679 (0.034)	0.528 (0.048)	0.222 (0.071)	0.246 (0.080)
*RPB*1	0.667 (0.057)	0.565 (0.053)	0.152 (0.043)	0.060 (0.061)

**Figure 4:**
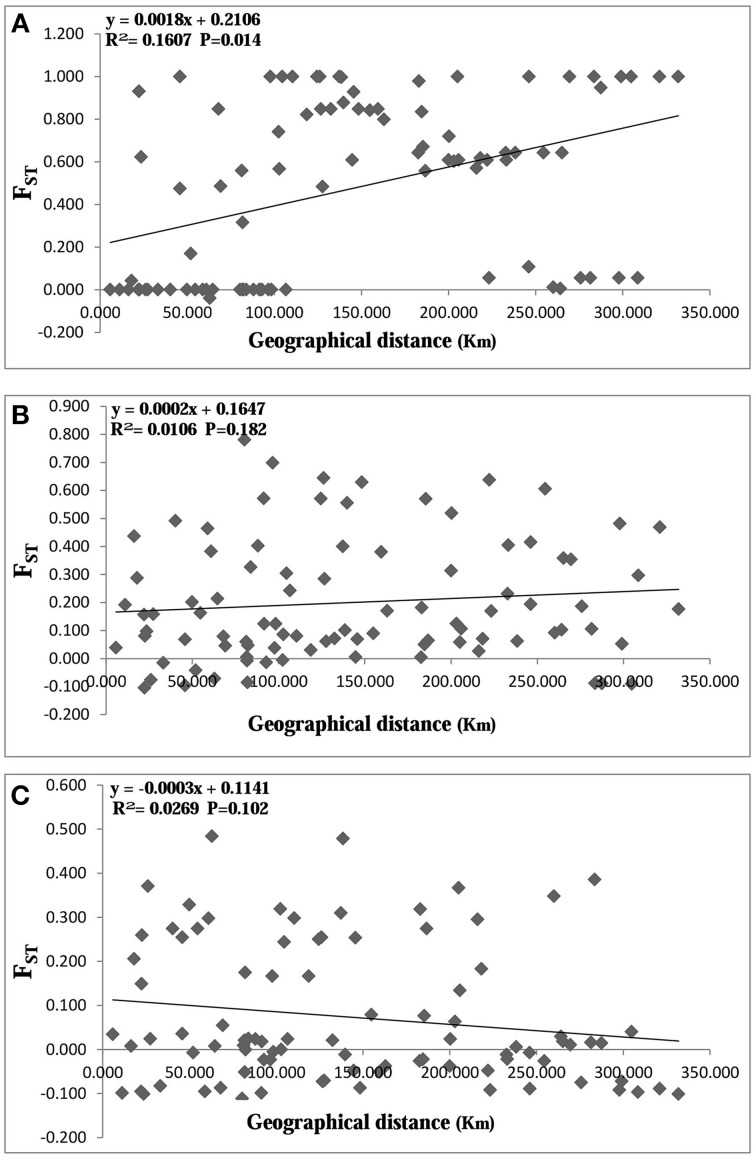
**Plot of geographical distance against genetic distance for populations of ***Cycas diannanensis*** inferred from cpDNA (A), nDNA ***SmHP*** (B), and nDNA ***RPB***1 (C)**.

AMOVA analysis revealed that 81.85% of the genetic variation was shared among populations and 18.15% within populations for cpDNA (Table 4), indicating a high level of genetic variation among populations. For nDNA, however, only 5.46 and 25.06% of the genetic variation was partitioned among populations, and 94.54%, 74.94% within populations for *RPB*1 and *SmHP* respectively, which showed low level of interpopulation genetic variation and high intra-population variation. Mantel test results showed significant effect (*R* = 0.401, *P* < 0.05) of isolation by distance (IBD) by combined cpDNA data, indicating a positive correlation between genetic and geographical distance, while both two nDNA markers showed non-significant (for *SmHP, R* = 0.103, *P*>0.05; for *RPB1, R* = −0.164, *P*>0.05) correlations between such distances (Figure 4).

**Table 4:** **Results of analysis of molecular variance (AMOVA) based on the combined cpDNA sequences and nuclear loci sequence data from populations of ***C. diannanensis*****.

**Markers**	**Source of variation**	**d.f**.	**Sum of squares**	**Variance components**	**Percentage of variation (%)**
cpDNA	Among populations	13	563.001	4.342	81.85
	Within populations	123	118.400	0.963	18.15
*SmHP*	Among populations	13	71.571	0.398	25.06
	Within populations	138	164.357	1.191	74.94
*RPB1*	Among populations	13	26.946	0.075	5.46
	Within populations	129	168.371	1.306	94.54

**Figure 5:**
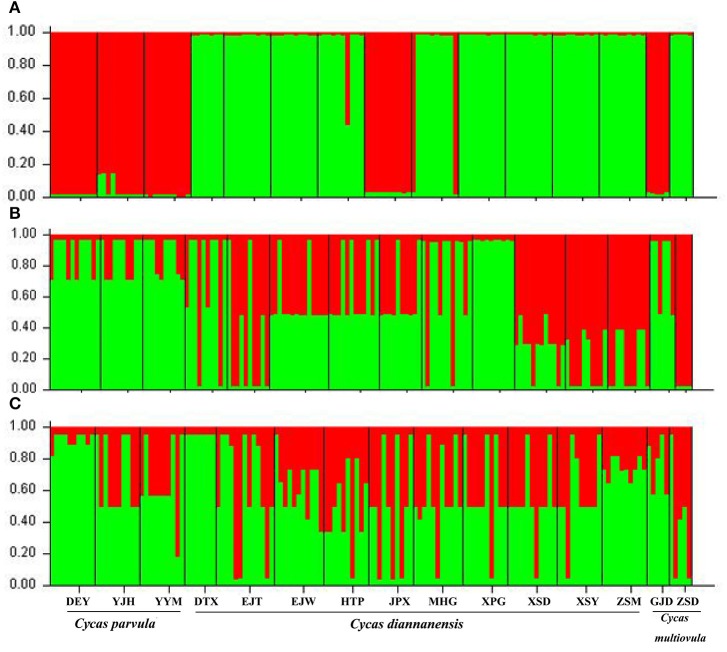
**Estimated genetic clustering (***K*** = 2 for all three markers) obtained with the STRUCTURE program from 15 populations of the three ***Cycas*** species based on cpDNA sequence (A) and nDNA ***SmHP*** (B), ***RPB***1 (C)**. Black lines separate different populations.

The STRUCTURE analysis which used the ΔK method based on a combined chloroplastic data in the whole 15 populations of the three species showed *K* = 2 was the optimal value (Figure S2A), dividing populations of the three sympatric species into two clusters: the first contained most *C. diannanensis* populations and one *C. multiovula* population, the other group included all the three *C. parvula* populations and one *C. multiovula* population (ZSD) as well as one *C. diannanensis* population (MHG). The results of two nDNA sequences also both suggested *K* = 2 (Figures S2B,C) a better solution than other K values, while no distinct genetic structure could be obtained from the two nuclear data set, with the disparate genetic components sharing in all different populations (Figure 5).

### Neutrality test, mismatch analysis and bayesian skyline reconstruction

The results of the Neutrality Test inferred from cpDNA showed a negative Tajima's D value and positive Fu and Li's F^*^ (see Table 5), which were both non-significant, implying the populations experienced no bottleneck effect or population expansion in history. The nuclear *SmHP* gene displayed both positive but non-significant value on Tajima's D and Fu and Li's F^*^, which was accorded with the combined cpDNA result. Whereas the nuclear *RPB*1 showed both positive Tajima's D value and Fu and Li's F^*^, which suggested historical bottleneck effect or genetic drift of the *Cycas* populations.

**Table 5:** **Parameters of neutrality tests and demographic analysis based on cpDNA and nDNA of ***C. diannanensis*****.

**Locus**	**Tajima's D**	Fu and Li's F^*^	**SSD**	**Raggedness**
cpDNA	−0.963	0.283	0.110^*^	0.184
*SmHP*	0.436	0.501	0.195	0.528
*RPB1*	3.275^**^	2.171^**^	0.214	0.485

Note:

* P < 0.05, significant difference;

** *P < 0.01, the most significant difference*.

**Figure 6:**
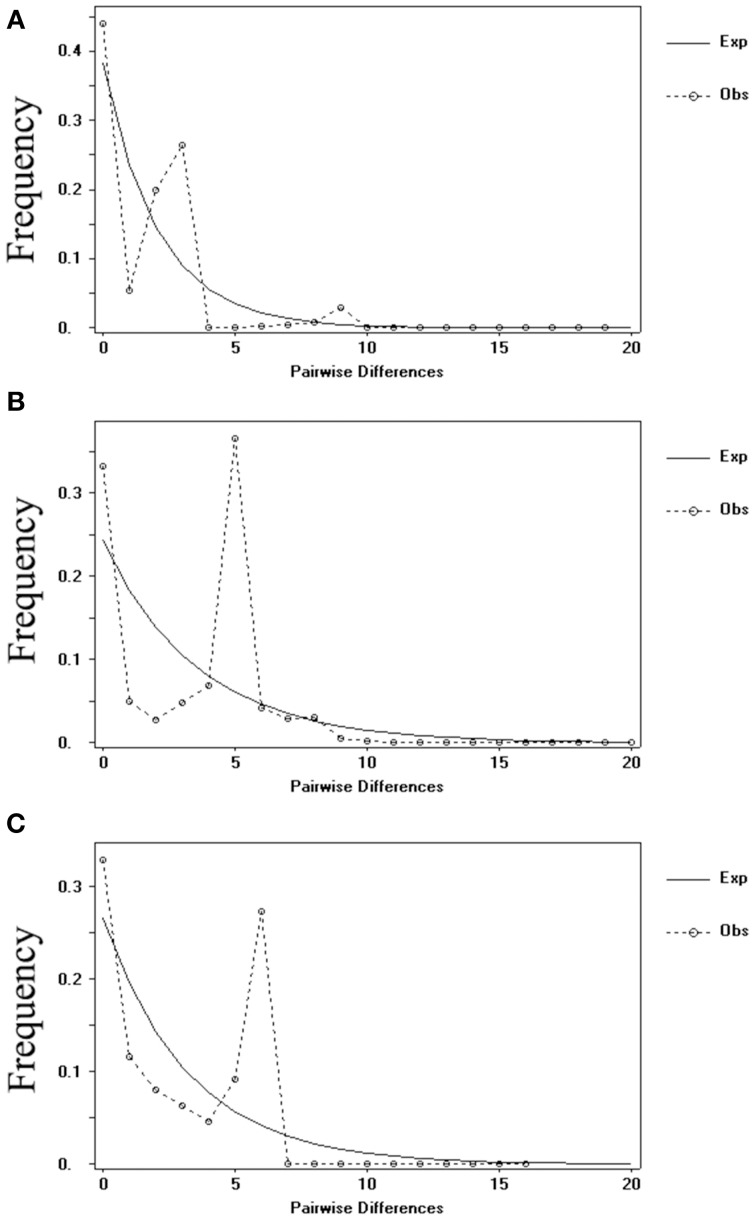
**Distribution of the number of pairwise nucleotide differences for cpDNA sequence (A) and nDNA ***SmHP*** (B), ***RPB***1 (C) sequences data in ***C. diannanensis*****. The solid line stands for expected values and the dashed line represents observed values under a model of sudden population expansion.

Meanwhile, the results of the mismatch analysis (Figure 6) for all populations displayed a multimodal distribution pattern with non-significant positive SSD and raggedness values for cpDNA and nDNA (*SmHP*), indicating these populations had not undergone a recent population expansion. While result inferred from nuclear *RPB*1 gene showed a unimodal curve, suggesting that population had experienced bottleneck events in history.

**Figure 7:**
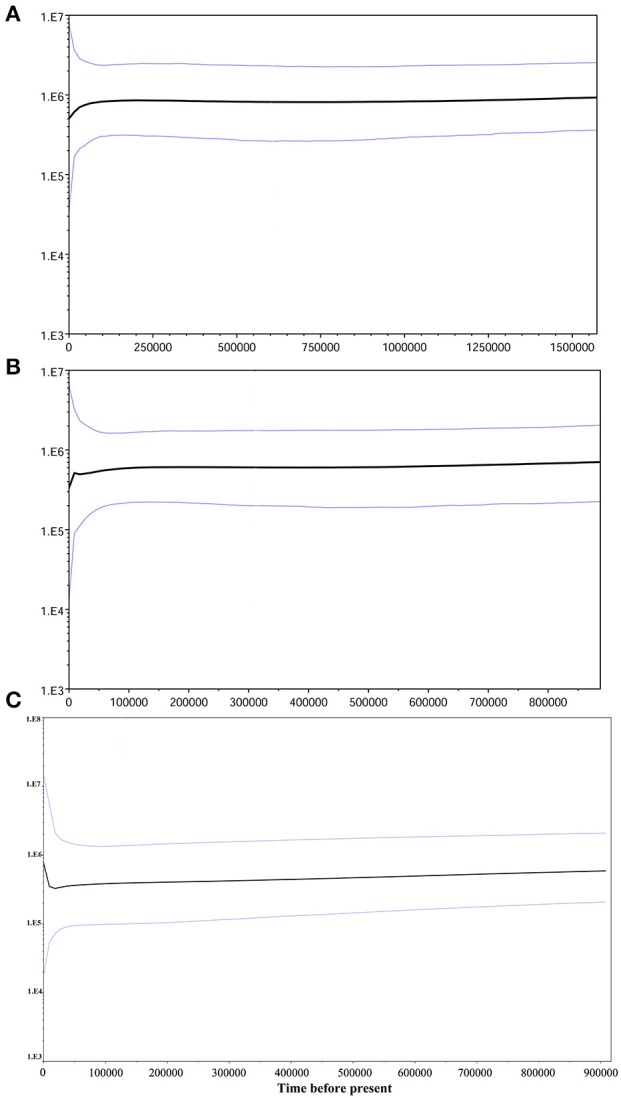
**Bayesian skyline plot based on cpDNA (A) and single copy nuclear gene ***SmHP*** (B) as well as ***RPB***1 (C) for the effective population size fluctuation throughout time**. Root height is set as median of the maximum time. Black line: median estimation; areas between gray lines: 95% confidence interval.

The skyline plots of historical population size dynamics analyzed by BEAST based on different datasets from the bayesian simulation were showed in Figure 7. The skyline plot indicated a long period of population equilibrium and recent declines (since 50–100 thousand years ago) in population size (Figure 7A, cpDNA; Figure 7B, nDNA *SmHP*) of the investigated populations during Quaternary glaciations. While for nDNA *RPB1*, a quite recent subsequent expansion after historical population decreasing (Figure 7C) was performed, which accorded with its possible bottleneck events in history detected by the above mismatch analysis.

## Discussion

### Species delimitation of the three *Cycas* species

Species delimitation is one of the two major goals of systematics (Wiens, 2007). As “no one definition has as yet satisfied all naturalists, yet every naturalist knows vaguely what he means when he speaks of a species” proposed by Darwin (Darwin, 1859), it has arouse explosive issues attempting to define what a species is and guide what we should take into account when determining this definition. In this study, we admit and adopt the unified species concept as a “lineage” (de Queiroz, 1998, 2005), which is separation of the theoretical concept of species (as separately evolving metapopulation lineages) (De Queiroz, 2007) and offered operational criteria for species delimitation.

Generally, shared haplotypes between different species might be given risen by hybridization which introduces new genes to other species, or incomplete linage sorting that retains ancestral haplotypes in the processing of speciation (Chiang et al., 2009). Under the tree-based criteria of species delimitation of haplotypes (Sites and Marshall, 2003), the detected lineages (DNA haplotypes) of the three species in this study (see Figures 2, 3) neither underwent sufficient isolation for coalescence to monophyly in cladogram or a “contiguous section” in the network analysis (CHA, Brower, 1999), nor for geographical character divergence (Wiens and Penkrot, 2002). Although our Structure analysis of cpDNA detected two distinct clusters from the 15 populations, the two lineages didn't correspond to the morphological characteristics or lineage aggregation, but partially corresponded to geography through genetic diversity. In addition, the widely shared haplotypes inferred from our nuclear data revealed possible hybridization or introgression within the three *Cycas* species, which blurred the specificity of the three species in history as well. For the populations or species which are forming their lineages, no such specificity is kept in evolution history, which brings about little unique genes throughout the genome, although they occupy morphological polymorphism. As the divergent time of the genus *Cycas* is quite recent (Nagalingum et al., 2011), as well as the young lineages of different haplotypes among the three species (Figure 3), we infer the reason for sharing haplotypes between populations may also contribute to the historical incomplete linage sorting of *Cycas*, resulting in the present non-monophyletic phylograms.

Meanwhile, these three species share overlapped distributions in the undergrowth habitat on mountain slopes of the Red River basin, making it possible for immigrations and gene flows among these populations (Figure 1). Gene flow might exist in form of continuous gene introgression within partial genes between some closely related species in the processing of speciation, which suggests the maintaining of reproductive isolation and morphological specificity as well as the ecological characters between different species. As a biparental inherited property, nuclear genes might be more suitable for species delimitation as its larger effect population size than cpDNA in plants (Comes and Kadereit, 1998), which is more difficult to be fixed in evolution process. Our nuclear data suggested the focal *Cycas* species could be a single and nonexclusive species as the paraphyletic and weak supported clades for aggregating populations, as well as the discordant geography for clusters (lineages) (see Wiens and Penkrot, 2002). Even though the phylograms between cpDNA and nDNA are inconsistent, the phylogenic tree of the three species all demonstrated scattered populations from *C. parvula* and *C. multiovula* nesting into the most *C. diannanensis* (Figure S1), indicating possible introgression between these species, which was also supported by the results of STRUCTURE based on nDNA data (Figures 5B,C). Thus, considering a comprehensive wild examination and specimen comparison from the herbarium which showed few and unobvious difference in morphology of the three species, as well as overlapped distribution, non-reproductive isolation, incomplete lineage sorting and possible introgression between the populations according to both chloroplastic and nuclear data analysis basing on adequate sampling in this study, we propose the three *Cycas* species along the Red River as one species, with *C. parvula* and *C. multiovula* incorporated into *Cycas diannanensis*.

### Taxonomy treatment and synonyms

*Cycas diannanensis* Z. T. Guan & G. D. Tao in Sichuan Forestry and Design, 1995(4): 1-2.

*C. pectinata* var. *manhaoensis* C. J. Chen & P. Yun, *Acta Bot. Yunnan*. 17(4): 400, 1995; *C. parvula* S. L. Yang & D. Y. Wang, *Cycads in China*. 93. 1996; *C. multiovula* D. Y. Wang, *Cycads in China*. 83. 1996.

### Genetic diversity and genetic structure of *C. diannanensis*

Genetic diversity could maintain the reproduction fitness adaptive evolution of species, which suggested low level of genetic variation might increase the possibilities of inbreeding and the risk of extinction (Lande, 1988; Reed and Frankham, 2001). We incorporated individuals from ZSD and ZSM populations from the same location together as one (ZSM), since they were regarded as the same species following our conclusion above. Our cpDNA result on *C. diannanensis* reveals it possesses higher haplotype diversity and total genetic diversity (*Hd* = 0.5642, *H*_*T*_ = 0.627) than *C. debaoensis* (*Hd* = 0.492, *H*_*T*_ = 0.564) (Zhan et al., 2011). However, in comparison with other reported *Cycas* species, it shows relatively lower level of diversity than *C. simplicipinna* (*Hd* = 0.846, *H*_*T*_ = 1) (Feng et al., 2014) and *C. revoluta* (*Hd* = 0.641, *H*_*T*_ = 0.641) (Kyoda and Setoguchi, 2010). Meanwhile, the total level of genetic diversity of *C. diannanensis* is lower than the mean value of 170 plant species that was estimated from cpDNA-based studies (*H*_*T*_ = 0.67) (Petit et al., 2005), and its haplotype diversity is lower than some endangered species such as *Hygrophila pogonocalyx* (*Hd* = 0.870) (Huang et al., 2005) and *Dysosma versipellis* (*Hd* = 0.924) (Qiu et al., 2009). For nuclear DNA, our data also displayed a relatively low level of total genetic diversity (for *RPB1*: *H*_*T*_ = 0.667, *SmHP*: *H*_*T*_ = 0.679) when compared with *C. simplicipinna* (*H*_*T*_ = 0.878, by ITS) (Feng et al., 2014), and other genetic diversity analysis inferred by nuclear genes, such as *Cardamine nipponica* (*H*_*T*_ = 0.689, 0.798, 0.885, three nuclear genes) (Ikeda et al., 2008), *Psammosilene tunicoides* (*Hd* = 0.724) (Zhang et al., 2011) and *Rhododendron pseudochrysanthum* (*Hd* = 0.881, Huang et al., 2011).

Generally, low level of genetic diversity occurs in the species that are rare, endangered or endemic, for their few and isolated populations as well as their adaptation in one-fold habitat (Spielman et al., 2004). Drift might be incidental with the populations with continuous distribution areas or low effective population size, which would lead to the low level of genetic diversity (Templeton et al., 2001; Marchelli et al., 2010). Most of the wild population sizes based on our survey, however, were less than 50 (Table 1), with higher haplotype diversity being detected in the populations with larger population size (such as MHG and HTP). However, species that actively migrates toward refuge areas can maintain higher levels of genetic diversity in refugia if their range contraction is rapid (Arenas et al., 2012), even though the species occupies isolated patches. Genetic drift and inbreeding within extremely small populations caused by habitat fragmentation (Young et al., 1996) could be the main reason for the low levels of genetic differentiation at nDNA among populations of *C. diannanensis* in this study. In addition, lower genetic diversity at cpDNA than nDNA in our study (Table 3) might be attributed to lower evolutionary rates as well as the uniparental property of chloroplastic genes which are more likely to be fixed (Hewitt, 2001).

As an ancient gymnosperm and woody plant species, cycads are considered to possess high genetic variations within populations and low level of differentiation among populations for their diecious habit and long life cycle for millions years of evolving genealogies. These characters make it possible for them to accumulate enough genetic variations for adaptation under the selective pressure from the historical geographical and climate events, and develop migrating strategies allowing them to track the most suitable environment (Hamrick et al., 1992; Arenas et al., 2012). In the case of our studied species, we detected a high genetic differentiation among populations through cpDNA data but relative low genetic differentiation with nDNA data (Table 4). This discordance might be explained by different inherited and dispersal patterns between cpDNA and nDNA in *C. diannanensis*. As the former one is maternally inherited in *Cycas* and dispersed only by seeds, whereas nDNA is biparentally inherited and owns both the ways of seeds and pollens, which offer opportunities for nDNA to obtain more genetic components among populations by gene flow. Moreover, recombination within nuclear genome may play another important role in gaining more potential genetic diversity of nDNA.

Significant genetic differentiation of *C. diannanensis* was detected on the basis of both cpDNA (*F*_*ST*_ = 0.819) and nDNA (*SmHP*: *F*_*ST*_ = 0.251; *RPB1*: *F*_*ST*_ = 0.055). Particularly, a distinct phylogeographical structure with cpDNA haplotypes distribution was revealed by the result of U test (*N*_*ST*_ > *G*_*ST*_, Table 3), which is corresponded with our IBD test result by Mantel test. The populations HTP, ZSM (ZSD), XSD, XSY, EJW, EJT, DTX, and XPG which overall occupied low genetic diversity distributed along the upstream “Ejia-Jiepai” basin, and populations DEY, YJH, YYM, GJD, MHG as well as JPX that owned high genetic diversity distributed along the downstream “Yuanjiang-Nanhun” basin (Figure 1A). As Jiepai that located in the middle of Red River fault zone is the turning point of the “neo-tectonic activity” (Oligo-Miocene, ~23Myr) after the collision of Indian Sub-continent with Laurasia (Tapponnier et al., 1990; Harrison et al., 1996), the gradually enhanced breakage activities from this location to south and north created the discrepancies in geology and climate condition between different drainages (Zhu et al., 2002), resulting in distinct habitats of extant distribution pattern of cpDNA haplotypes.

### Population historical dynamics of *C. diannanensis*

Glaciations, especially Pleistocene glaciations made deep effects on the spatial distribution of plants (Hewitt, 2000). These sessile organisms are thought to have different response scenarios during the Quaternary ice age, mostly choosing to shift their latitude or altitude ranges (Davis and Shaw, 2001) or seeking for a “shelter” (refugium hypothesis, Holder et al., 1999). Previous studies mostly showed species expansion or stability during the Last Glacial Maximum (LGM) (Marko et al., 2010; Bisconti et al., 2011; Cunha et al., 2011). Within gymnosperms, some species such as *Taxus wallichiana* (Liu et al., 2013), *C. revoluta* and *C. taitungensise* (Chiang et al., 2009) also expanded their geographical distribution during the ice age, while with some other *Cycas* species (e.g., *C. debaoensis* Zhan et al., 2011 and *C. simplicipinna* Feng et al., 2014), a contraction process pattern were detected. From a two set genetic data (cpDNA and nDNA *SmHP*) of three markers in this study, a possible similar population contraction may appear in *C. diannanensis* in history from the results of Bayesian skyline plots (Figures 7A,B). Mismatch analysis of the above two data set also rejected the population expansion hypothesis (Figures 6A,B) but a population contraction or a population dynamic equilibrium. However, the nuclear gene *RPB*1 provided unexpected result in the populations of *C. diannanensis* which showed a small recent expansion after long term of declining (bottleneck effect) by Bayesian skyline and the possible bottleneck events deducted from mismatch analysis. This discordance may be attributed to historical genetic drift or larger selective pressure existed on this gene (Figures 6C, 7C). Meanwhile, as larger genetic loss may be induced by slower range contraction or shift which brings about lower level of genetic diversity (Arenas et al., 2012), we argue that *C. diannanensis* were previously widely and continuously distributed before the ice age and slowly contracted (also revealed by our skyline plots of *RPB1*) into several isolated surviving populations during the glaciation, with relative lower genetic diversity being detected in this study.

It might be suspicious with the reported *Cycas* species distributed in southwest China had all experienced population retreats (Zhan et al., 2011; Feng et al., 2014, this study) rather than expansion during the Quaternary glaciation. In the case of *Cycas diannanensis*, the historical dynamics might tend to be closely related with the disjunctive distribution in the “Yuanjiang-Nanhun” basin and “Ejia-Jiepai” basin in the Red River fault zone at present. The Red River fault zone, a geographical boundary of South-China plate and Indo-Sunda plate as well as the principal displacement zone between the South-China plate and Indo-China Peninsula (Zhu et al., 2003), underwent frequent historically geological activities and climate changes since late Miocene. The most recent two dextral strike slip fault events occurred at 5.5 ± 1.5 MaBP and 2.1 ± 0.8 MaBP respectively (Xiang et al., 2007). Interestingly, the above timings were roughly accorded with the time of diversification of extant *Cycas* (~8.68MaBP, Nuclear plus Plastid gene, full sampled, Nagalingum et al., 2011) and the divergence of *Cycas diannanensis* haplotypes (2.0–3.8MaBP, this study). The fault region harbors numerous of *Cycas* species in its ranges, especially in the dry hot valley of southwest China (Wang et al., 1996), which can be considered as a typical glacial refugium for many plant species during Quaternary glaciation period. Therefore, it is possible that frequent geological activities in the Red River fault zone, impacts of glacial falling temperature (Harrison et al., 2001) as well as recent human activities (see discussion below) all exerted profound influences on the population dynamics (contraction) of *C. diannanensis*.

### Conservation implications for *C. diannanensis*

The main purpose of conservation genetics is to maintain the evolutionary ability of species for their adaptation to the varying environment (Frankham et al., 2002). The genetic constitution of one species is not only applied for distinguishing it from other species, but also determining its potential adaptation to the environmental variable changes (Van Dyke, 2008). Therefore, the conservation of species' genetic diversity is critical for its long-term survival (Schemske et al., 1994). Our chloroplastic and nuclear data that revealed low genetic diversity as well as the declining population size may trace the species' endangering status. Meanwhile, reduced allelic richness may limit a species' ability to respond to changing selection pressures (Frankel, 1995; Young et al., 1996). In the case of *C. diannanensis*, most populations occupied one specific haplotype, thus it may be risky as it can lead to a loss of adaptability once they are confronting with climate change or external biological disturbance.

Cycads, for their palm-like leaves and abundant symbols in tradition, are often cultivated as ornaments or traded for medical value (Cousins et al., 2011, 2012). For such anthropogenic reasons, the population size of wild cycads decreases extremely in recent decade years and most cycad species are classified in the Red List (IUCN, 2015). *Cycas diannanensis* is distributed in a narrow region along the “Ejia-Jiepai” basin and “Yuanjiang-Nanhun” basin in the Red River fault zone, where are often accompanied with fragmented original habitats, and disturbed by human activities such as plowing and grazing. To restore genetic diversity loss resulting from such landscape fragmentation, it needs to be maintained over dozens or hundreds of generations of the endangered species to have a significant effect on the local genetic diversity and population structure (Mona et al., 2014). Furthermore, for the purpose of protecting enough genetic components of *C. diannanensis*, a strategy of *in-situ* and *ex-situ* conservation should be taken, especially with the populations harboring relative higher diversity such as downstream populations HTP, YJH, YYM, MHG, and GJD. Simultaneously, for the populations DEY and JPG which possess high genetic distance and unique haplotypes, measures should also be adopted in order to protect the whole genetic diversity. Considering the above two populations (DEY, JPG) are conserved by cultivation in local regions, seed or seedling reproduction from the two populations are suggested to artificially introduce the genetic components into other regions or in the wild to increase the individual number and genetic diversity of each population.

## Availability of supporting data

The data set of the DNA sequencing data in our study are deposited in GenBank under accession numbers KT334601-KT334653.

## Conflict of interest statement

The authors declare that the research was conducted in the absence of any commercial or financial relationships that could be construed as a potential conflict of interest.

## Supplementary material

Figure S1 **Phylograms of all lineages from the three *Cycas* species inferred from Bayesian inference based on combined cpDNA (A) and single copy nuclear gene *SmHP* (B) as well as *RPB*1 (C) with *C. tanqingii* being employed as outgroup**. Number on each node stands for posterior probability (PP).

Figure S2 **Delta-K curves by Structure Harvester based on the Structure analysis of cpDNA (A) and single copy nuclear gene *SmHP* (B) as well as *RPB*1 (C)**.
