# Supplementary figures and images for: Species delimitation, genetic diversity and population historical dynamics of Cycas diannanensis (Cycadaceae) occurring sympatrically in the Red River region of China
Source: Front Plant Sci. 2015 Sep 8;6:696. doi: 10.3389/fpls.2015.00696 (PMC4562272; doi:10.3389/fpls.2015.00696)

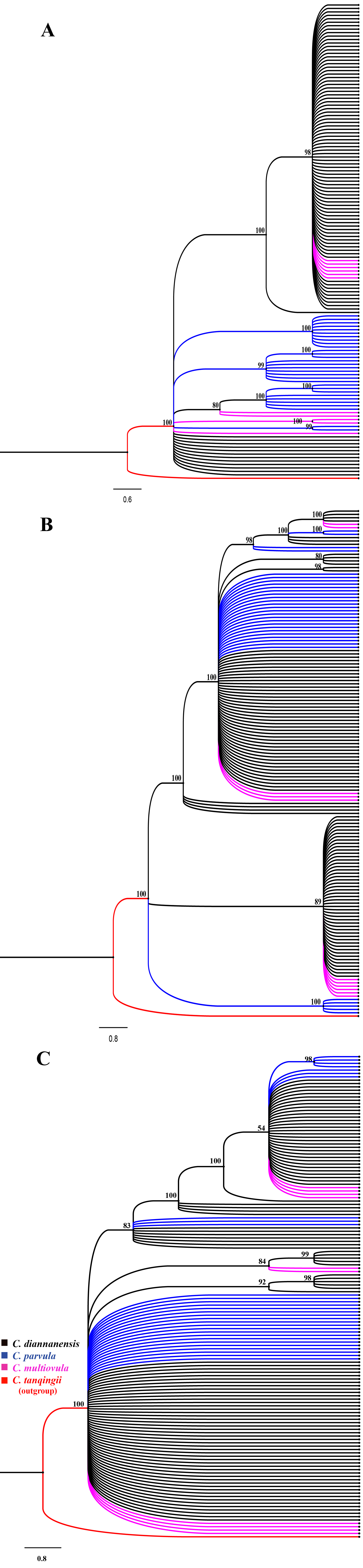

Supplement: Figure S1 — Phylograms of all lineages from the three Cycas species inferred from Bayesian inference based on combined cpDNA (A) and single copy nuclear gene SmHP (B) as well as RPB1 (C) with C. tanqingii being employed as outgroup. Number on each node stands for posterior probability (PP). [file Image1.JPEG]

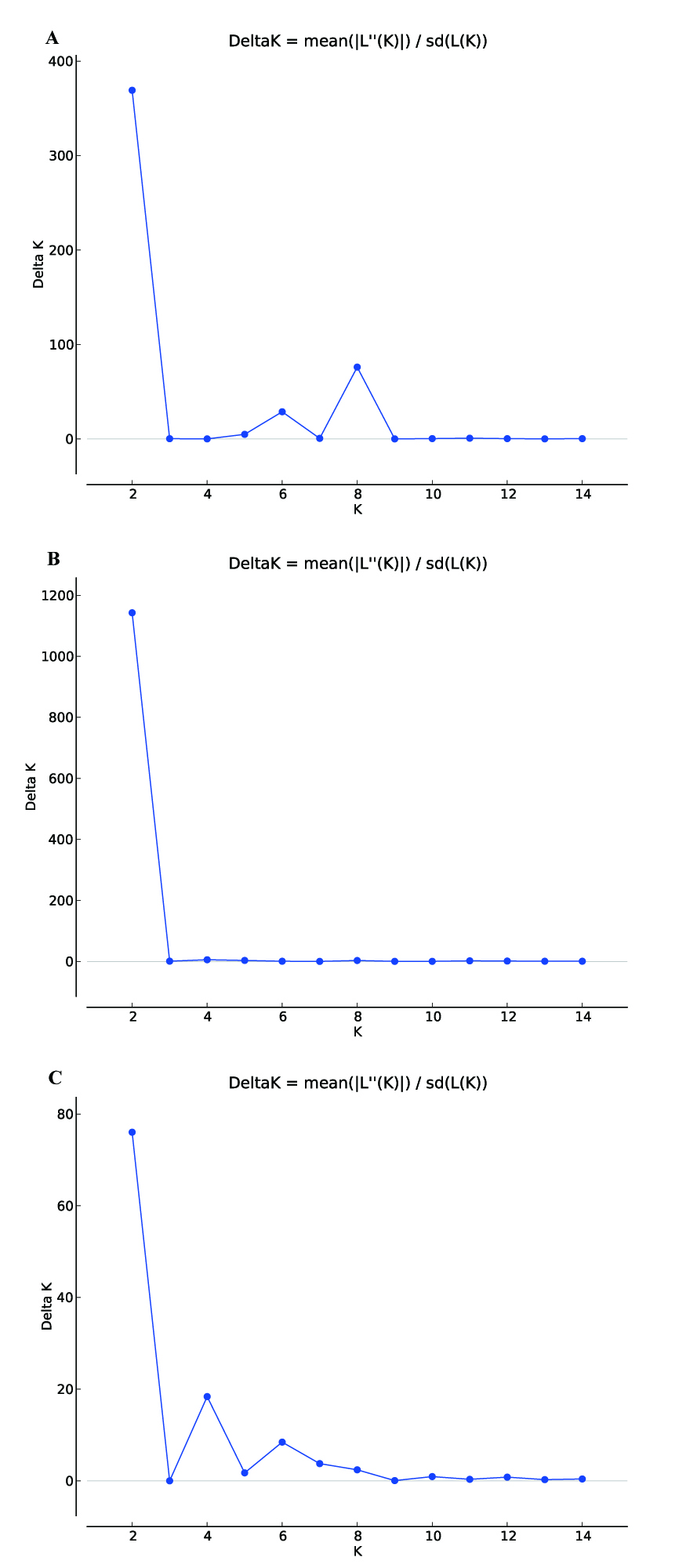

Supplement: Figure S2 — Delta-K curves by Structure Harvester based on the Structure analysis of cpDNA (A) and single copy nuclear gene SmHP (B) as well as RPB1 (C). [file Image2.JPEG]
