# Supplementary material for: Species delimitation, genetic diversity and population historical dynamics of Cycas diannanensis (Cycadaceae) occurring sympatrically in the Red River region of China
Source: Front Plant Sci. 2015 Sep 8;6:696. doi: 10.3389/fpls.2015.00696 (PMC4562272; doi:10.3389/fpls.2015.00696)
Supplement: Supplementary file 3 [file Table1.DOCX]

**Table S1.** The composition of haplotypes, haplotype diversity (*Hd*) and nucleotide diversity (Pi) calculated in combined cpDNA sequences and nDNA sequences of *C. diannanensis* investigated in this study.

| Population  code | cpDNA | | | *SmHP*(F004-R725) | | | *RPB*1(F010-R1142) | | |
| --- | --- | --- | --- | --- | --- | --- | --- | --- | --- |
|  | Haplotypes | *Hd* | Pi (10^3^) | Haplotypes | *Hd* | Pi (10^3^) | Haplotypes | *Hd* | Pi (10^3^) |
| DEY | H1(10) | 0 | 0 | S1(2) S2(9) | 0.327 | 3.94 | R1(3) R2(7) | 0.467 | 0.51 |
| DTX | H2(7) | 0 | 0 | S2(4) S3(3) S4(2) | 0.722 | 4.35 | R2(7) | 0 | 0 |
| EJT | H2(10) | 0 | 0 | S2(4) S3(7) | 0.509 | 3.83 | R2(7) R3(5) R4(1) | 0.603 | 3.40 |
| EJW | H2(10) | 0 | 0 | S2(8) S3(5) | 0.513 | 3.86 | R1(1) R2(4) R3(1) R5(4) R6(1) | 0.782 | 2.67 |
| GJD | H3(1) H4(2) H5(2) | 0.800 | 3 | S2(3) S3(1) S5(2) | 0.733 | 5.32 | R2(2) R7(2) R8(1) | 0.800 | 2.41 |
| HTP | H2(9) H6(1) | 0.200 | 1 | S2(7) S3(5) | 0.530 | 4.00 | R2(2) R3(3) R9(5) | 0.689 | 2.83 |
| JPX | H4(10) | 0 | 0 | S2(7) S3(4) | 0.509 | 3.83 | R2(5) R3(5) | 0.556 | 3.65 |
| MHG | H2(8) H4(1) H7(1) | 0.378 | 0.46 | S2(3) S3(3) S5(2) S6(3) | 0.818 | 5.42 | R1(1) R2(5) R3(5)) | 0.636 | 3.59 |
| XPG | H2(10) | 0 | 0 | S2(8) S5(2) S7(1) | 0.473 | 2.25 | R2(6) R3(4) | 0.533 | 3.51 |
| XSD | H2(10) | 0 | 0 | S2(1) S3(6) S8(4) | 0.618 | 3.01 | R2(6) R3(5) | 0.545 | 3.59 |
| XSY | H2(10) | 0 | 0 | S3(9) S9(1) | 0.200 | 1.20 | R2(6) R3(4) | 0.533 | 3.51 |
| YJH | H8(2) H9(2) H10(6) | 0.622 | 0.18 | S1(2) S2(8) | 0.356 | 4.28 | R2(7)R3(3) | 0.467 | 3.07 |
| YYM | H11(5) H12(3) H13(2) | 0.689 | 1.79 | S1(1) S2(6) S10(2) S11(2) | 0.691 | 4.22 | R2(6) R10(4) | 0.533 | 2.92 |
| ZSM  (ZSD) | H2(15) | 0 | 0 | S3(12) (ZSD,ZSM)  S12(3) (ZSM) | 0.343 | 2.07 | R1(5) R11(5) (ZSM)  R2(2) R3(3) (ZSD) | 0.771 | 1.83 |
| Total |  | 0.564 | 0.87 |  | 0.670 | 4.71 |  | 0.671 | 3.02 |
